# Molecular characterization and genetic diversity of parvoviruses prevalent in cats in Central and Eastern China from 2018 to 2022

**DOI:** 10.3389/fvets.2023.1218810

**Published:** 2023-07-31

**Authors:** Shunshun Pan, Ruiqi Jiao, Xin Xu, Jun Ji, Ge Guo, Lunguang Yao, Yunchao Kan, Qingmei Xie, Yingzuo Bi

**Affiliations:** ^1^Henan Provincial Engineering and Technology Center of Animal Disease Diagnosis and Integrated Control, Henan Key Laboratory of Insect Biology in Funiu Mountain, Nanyang Normal University, Nanyang, China; ^2^Guangdong Provincial Laboratory of Lingnan Modern Agricultural Science and Technology, College of Animal Science and Veterinary Medicine, South China Agricultural University, Guangzhou, China

**Keywords:** feline panleukopenia virus, canine parvovirus type-2, parvovirus, phylogeny, mutation

## Abstract

Cats are a potential source of genetic diversity for parvoviruses. Herein, 134 samples were collected from cats with clinical gastroenteritis and analyzed for the presence of viral DNA via polymerase chain reaction, which revealed 48 positive samples. Identity analysis of VP2 nucleotide sequences indicated that these 48 strains, belonging to feline panleukopenia virus (FPV) and canine parvovirus type-2 (CPV-2; including new CPV-2a and CPV-2c genotypes), shared 94.59–99.94% nucleotide identity with the reference strains. The FPV strain F8 (isolated from Vietnam) appeared to be a recombinant of strains HB2003 and JS1901, whereas the Chinese CPV-2b strain BM-(11) isolated in 2011 was believed to be a recombinant of strains AH2008 and JS1901. In phylogenetic tree analysis based on VP2 nucleotide sequences, all obtained FPV strains and most reference FPV strains were clustered together, except strain BJ-22, which originated from monkeys. Further, two new CPV-2a strains (AH2005 and AH2008) were close to the newly reported Chinese CPV-2a strains but were distant from the other CPV-2a strains, namely CPV-339 (from the United States) and K022 (from South Korea). Additionally, the FPV and CPV-2 strains had high mutation rates in the antigenic regions of the VP2 protein. According to model prediction of the CPV–VP2 protein, these mutations may cause changes in the tertiary structure of VP2. The findings of this study can be used to improve the pre-evaluation of vaccination efficacy against diseases caused by FPV and CPV-2 in domestic cats and understand their genotypic transmission and mutation trends.

## 1. Introduction

Parvoviruses are nonenveloped single-stranded DNA viruses that infect various animals (1). Feline panleukopenia virus (FPV) and canine parvovirus type-2 (CPV-2) are members of the genus *Protoparvovirus* belonging to the family *Parvoviridae* (2). FPV and CPV-2 can cause severe intestinal diseases in cats and dogs with high mortality and infectivity (3). They are mainly transmitted through feces, urine, and oral secretions from infected animals and can cause severe symptoms such as increased body temperature, vomiting, viral diarrhea, and a marked decrease in leukocyte count (4). Since the early 20th century, FPV has been recognized as one of the main pathogens that cause feline viral diarrhea (5). In general, cats infected with FPV have high morbidity and mortality, and kittens aged <3 months are highly susceptible to this virus (6, 7). Further, CPV-2, which was discovered in the 1970s (8), causes a highly contagious and fatal disease in dogs (2). CPV-2 was previously believed to be evolved from FPV in domestic cats after cross-species transmission to wild carnivores as a viral intermediate (9), but the directionality of the mutations was not considered (10, 11). The genome coding region of FPV/CPV-2 contains two major expression cassettes, encoding nonstructural (NS1 and NS2 for the left open reading frame) and structural (VP1 and VP2 for the right open reading frame) proteins (12). VP2 is a significant component of the FPV/CPV-2 capsid protein and represents approximately 90% of the virion (11). It is a critical protein that determines the antigenic properties, host range, and receptor binding of FPV/CPV-2 (13). Multiple genotypes have been reported owing to the antigenic differences in VP2 across several CPV-2 strains. The genotypes of these strains are classified based on the key amino acid (aa) residues of VP2. The key aa residues of VP2 in different genotypes are as follows: classical CPV-2 containing 87Met, 101Ile, 300Ala, 305Asp., and 375Asn; CPV-2a containing 426Asn and 297Ser; CPV-2b containing 426Asp and 297Ser; the new CPV-2a strain containing 426Asn and 297Ala; the new CPV-2b strain containing 426Asp and 297Ala; CPV-2c containing 426Glu and 297Ala; and FPV containing 80Lys, 93Lys, 103Val, 323Asp., and 568Ala (14).

Initially, CPV-2 could not replicate in cats; however, currently, all CPV variants (CPV-2a to 2c) can infect cats and cause subclinical disease or feline panleukopenia (10). This study aimed to understand the evolution of FPV/CPV-2 circulating in domestic cats in Central and Eastern China. Samples collected from domestic cats were subjected to FPV/CPV-2 screening and molecular characterization of VP2.

## 2. Materials and methods

### 2.1. Sample preparation and viral DNA/RNA extraction

A total of 134 rectal swabs or fecal samples were collected from cats with clinical gastroenteritis at pet hospitals in Henan, Anhui, Jiangsu, and Hubei Provinces. Each sample was suspended in phosphate-buffered saline, and viral DNA/RNA was extracted using EasyPure Viral DNA/RNA Kit (TransGen Biotechnology, Inc., Beijing, China) according to the manufacturer’s instructions. The extracted viral DNA/RNA was stored at −80°C until analysis.

### 2.2. VP2 sequencing

For FPV/CPV-2, a pair of primers (targeting the nucleotides 2,761–4,536 according to the FPV strain HF1; accession number: MT614366) was used to amplify *VP2* via polymerase chain reaction (PCR), as described previously (15). The amplicons from each positive sample were ligated into the pMD18-T easy vector, and the recombined plasmids were sequenced by Syn-Biotechnology (Syn-Biotechnology, Suzhou, China). Clinical information on the sources of these sequenced parvoviruses is provided in Supplementary Table S1.

### 2.3. Identity, recombination, and phylogenetic analyses

Sequences were assembled using the SeqMan module of Lasergene 7 (DNASTAR, Inc., Madison, WI, United States). To display the results more intuitively, BioAider Version 1.314 was used to analyze the 48 strains and 41 reference strains (Supplementary Table S2) for differences, and the results were annotated using Chiplot online software^1^ (16).

Recombination of strains obtained in this study and reference strains was predicted using RDP 4.97.0.1 (17), with the default settings. The prediction methods included RDP (18), GENECONV (19), BOOTSCAN (20), MAXCHI (21), and CHIMERA (22), which were associated with *p*-values. The predicted recombination events were further validated using Simplot software Version 3.5.1 (23).

Based on complete VP2 nucleotide sequences, a phylogenetic tree was constructed using MEGA-X Version 10.1 (bootstrap replicates = 1,000) via the maximum likelihood method (Tamura 3 parameter model) (24).

### 2.4. Mutation sites, antigen, and tertiary structure prediction

The aa variations in the obtained and reference strains were predicted as potentially dominant B cell antigen epitopes via DNAMAN Version 5.2.2 (Lynnon Biosoft, Canada). The 11 obtained CPV-2 strains were compared with the original CPV-2 strain (790,312 strain; accession number: M38245). The classical and variant CPV-2 strains were structurally modeled using homology via SWISS-MODEL^2^ and were visualized using PyMOL Molecular Graphics System.

## 3. Results

### 3.1. Detection and genotypes of parvoviruses

The PCR assay revealed that 48 samples (35.82%, 48/134) were positive for FPV or CPV-2, and FPV accounted for 77.08% (37/48) of the positive samples. The obtained FPV or CPV-2 strains were confirmed to contain full-length VP2 genes (1755 bp). The CPV-2 strains included two new 2a strains from Henan and Jiangsu Provinces and nine CPV-2c strains from Henan Province.

### 3.2. Nucleotide identity of the VP2 gene

**Figure 1:**
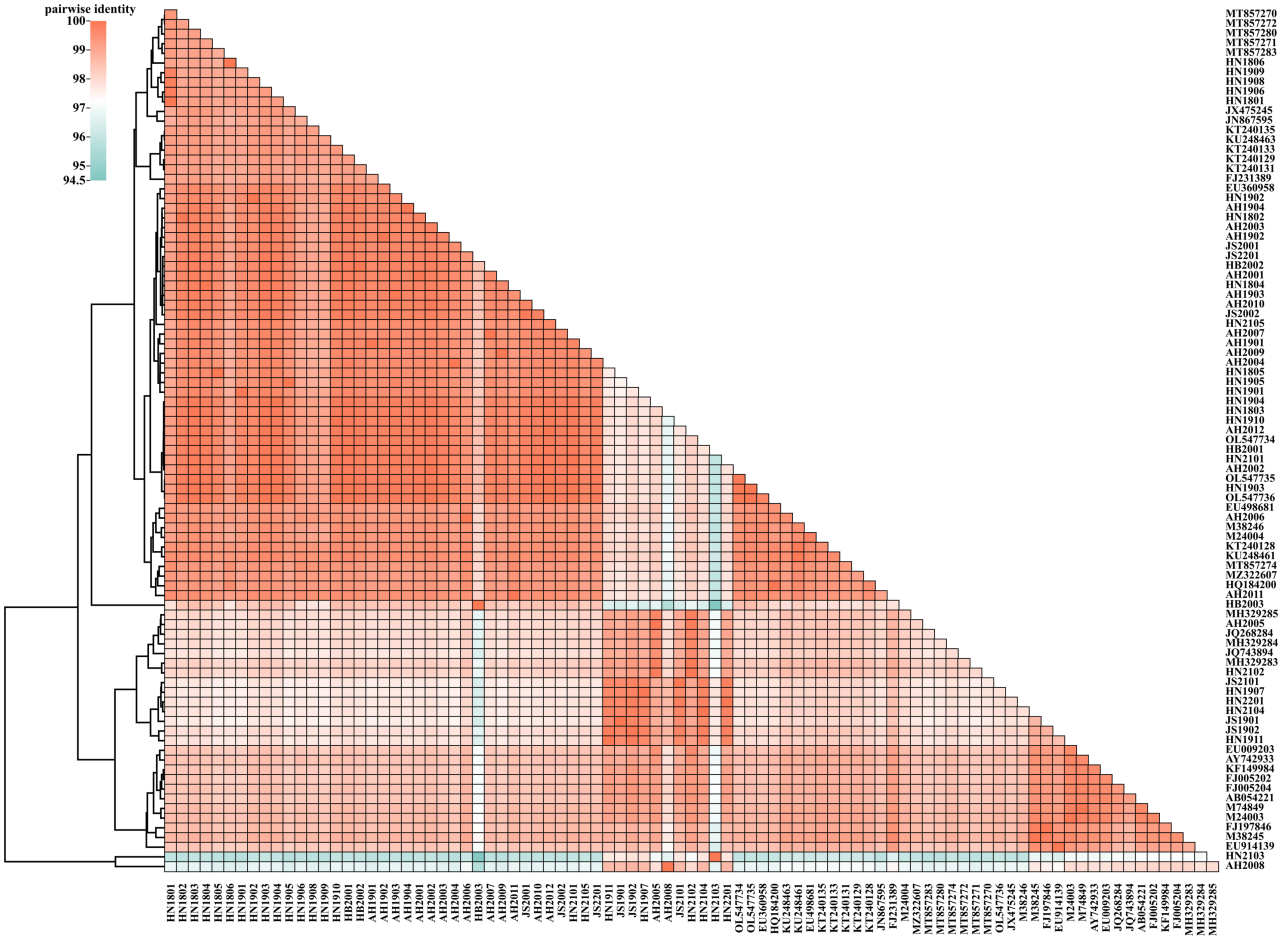
Two-dimensional pairwise identity plot of VP2 sequences of 89 strains constructed using BioAider V1.314. The grids of different colors indicate different identities, which were analyzed via clustering.

The VP2 nucleotide sequences of the 48 obtained strains and 41 reference strains were aligned and cluster-analyzed (Figure 1). The results revealed that all strains shared 94.59–99.94% nucleotide identity with each other; the FPV strains showed 97.72–99.94% identity, whereas the CPV-2 strains displayed 96.7–99.89% identity. No evident differences were noted in strain sequences across different provinces or years.

### 3.3. Recombination analysis

**Figure 2:**
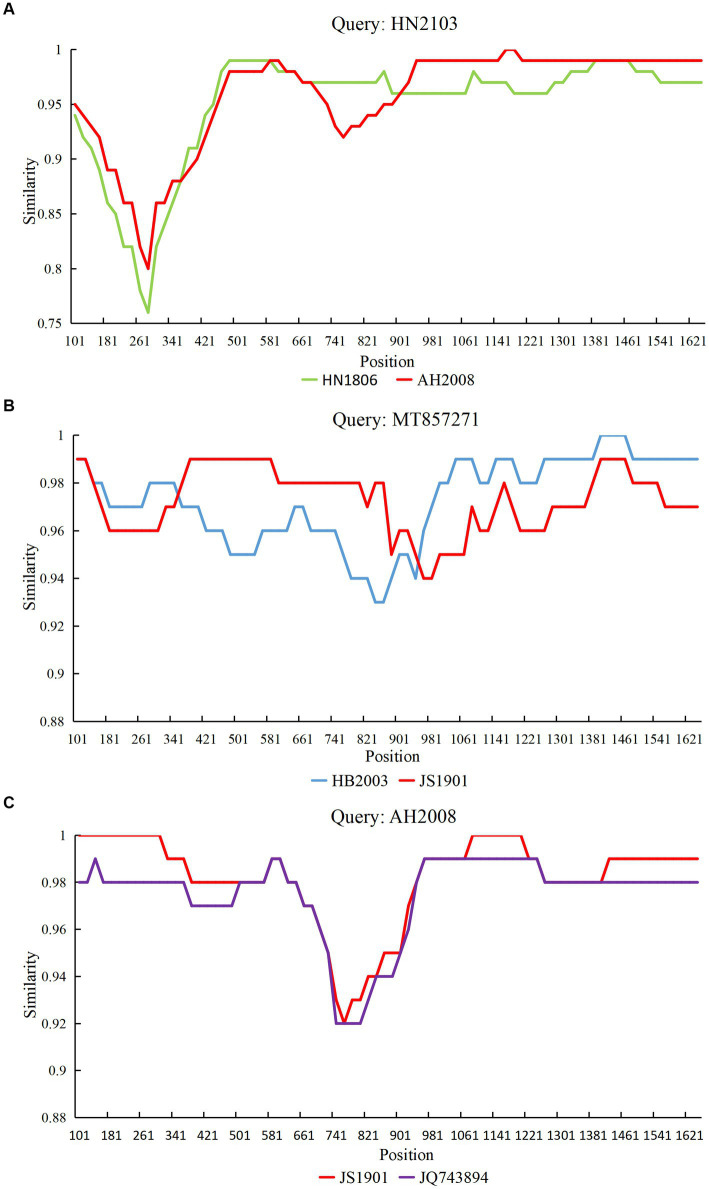
Simplot analysis of the variant strains obtained from three recombination events. The recombination breakpoint is indicated by the intersection point of the two lines. **(A)** Strain HN2103 was a recombinant of strains HN1806 and AH2008. **(B)** Strain MT857271 was a recombinant of strains HB2003 and JS1901. **(C)** Strain AH2008 was a recombinant of the strain JS1901 and reference strain JQ743894.

Based on RDP analysis, three major recombination events (1–3) were identified. Simplot software was used to analyze the recombination events. The results were consistent with those of RDP4 (Figure 2). The HN2103 strain (accession number: OQ868531; collected in 2021) was a recombinant of HN1806 (accession number: OQ868538; collected in 2018) and AH2008 (accession number: OQ868527; collected in 2020) strains in recombination event 1. In recombination event 2, HB2003 (accession number: OQ868569; collected in 2020) and JS1901 (accession number: OQ868523; collected in 2019) strains were the precursors of the reference FPV strain F8 from Vietnam (accession number: MT857271; collected in 2018). The AH2008 strain (collected in 2020) was a recombinant of JS1901 (accession number: OQ868523; collected in 2019) and BM-(11) (accession number: JQ743894; collected in 2011) strains in recombination event 3.

### 3.4. Phylogenetic analysis of FPV and CPV

**Figure 3:**
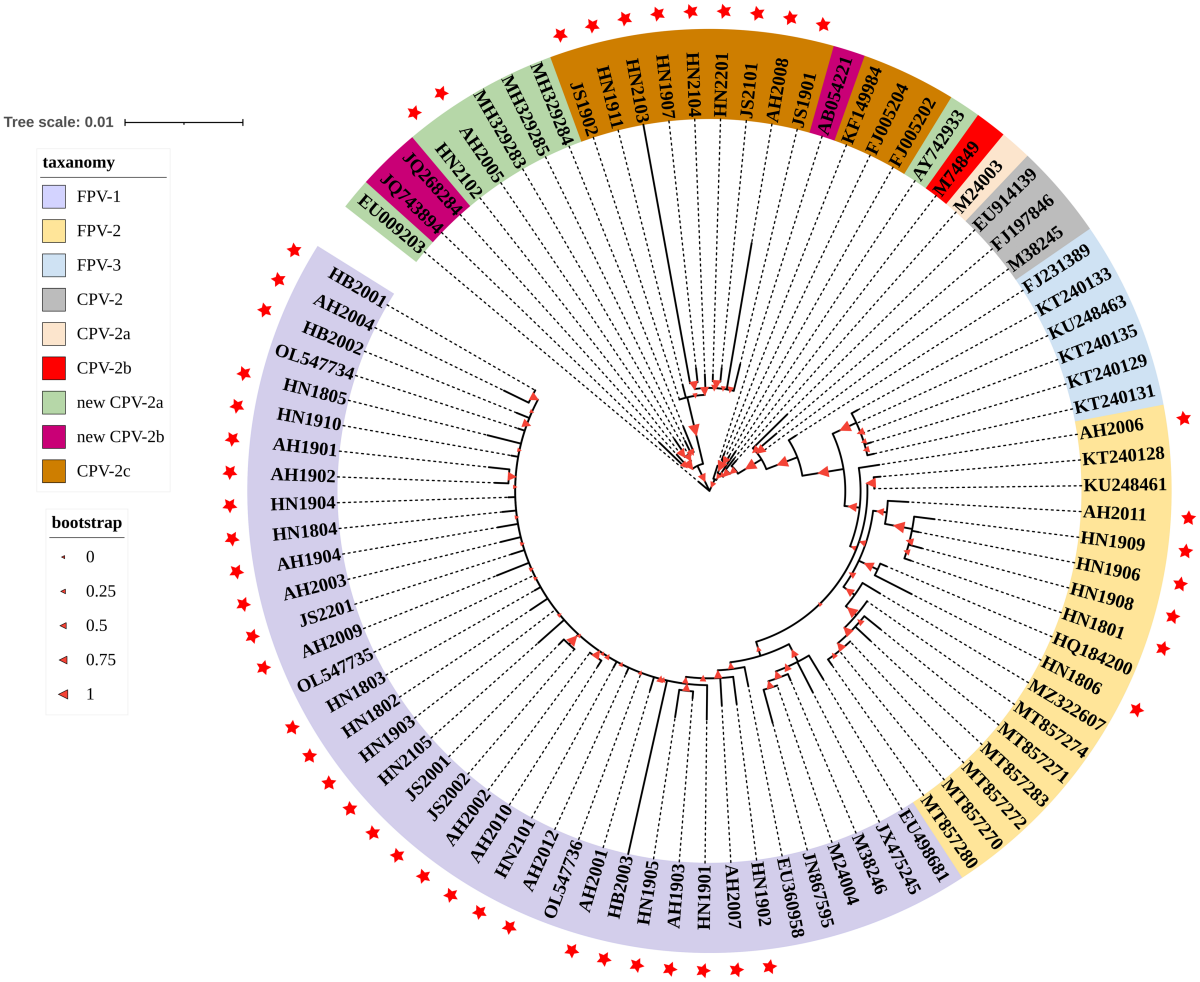
A phylogenetic tree based on the VP2 gene from the strains obtained in this study and reference strains. The 48 strains obtained from domestic cats in this study are indicated with a red star. Bootstrap values calculated from 1,000 replicates are indicated by the size of the red triangle on the respective branches. Different colors represent clade taxanomy of strains and the distance scale of the branch of the phylogenetic tree.

The evolutionary tree of parvovirus was constructed based on the VP2 nucleotide sequences of 89 FPV and CPV-2 strains (Figure 3). The FPV strains obtained in this study were grouped into three phylogenetic clusters and were closely related to most reported FPV strains but were distant from the isolated BJ-22 strain, which originated from a monkey with diarrhea (accession number: FJ231389). Based on the evolutionary relationship, the nine obtained CPV-2c strains were distant from the reported reference CPV-2c strains from Germany (G367-97 strain, accession number: FJ005202 and G333-99 strain, accession number: FJ005204) and Ecuador (2c-ME28-ECU2012 strain, accession number: KF149984). Two new CPV-2a strains (AH2005, accession number: OQ868526 and AH2008, accession number: OQ868527) obtained in this study were close to certain new CPV-2a strains from China but were distant from the new CPV-2a strains from the United States (CPV-339 strain; accession number: AY742933) and South Korea (K022 strain; accession number: EU009203).

### 3.5. Antigenic epitope predictions

To provide an immune-response base for FPV and CPV, the B cell epitope prediction of VP2 was performed in these 89 strains, revealing the presence of 20 potential linear epitopes (Supplementary Table S3). The epitope with the highest score (1.207) was located at the aa residue position of 247–256.

### 3.6. Mutation site analysis

**Figure 4:**
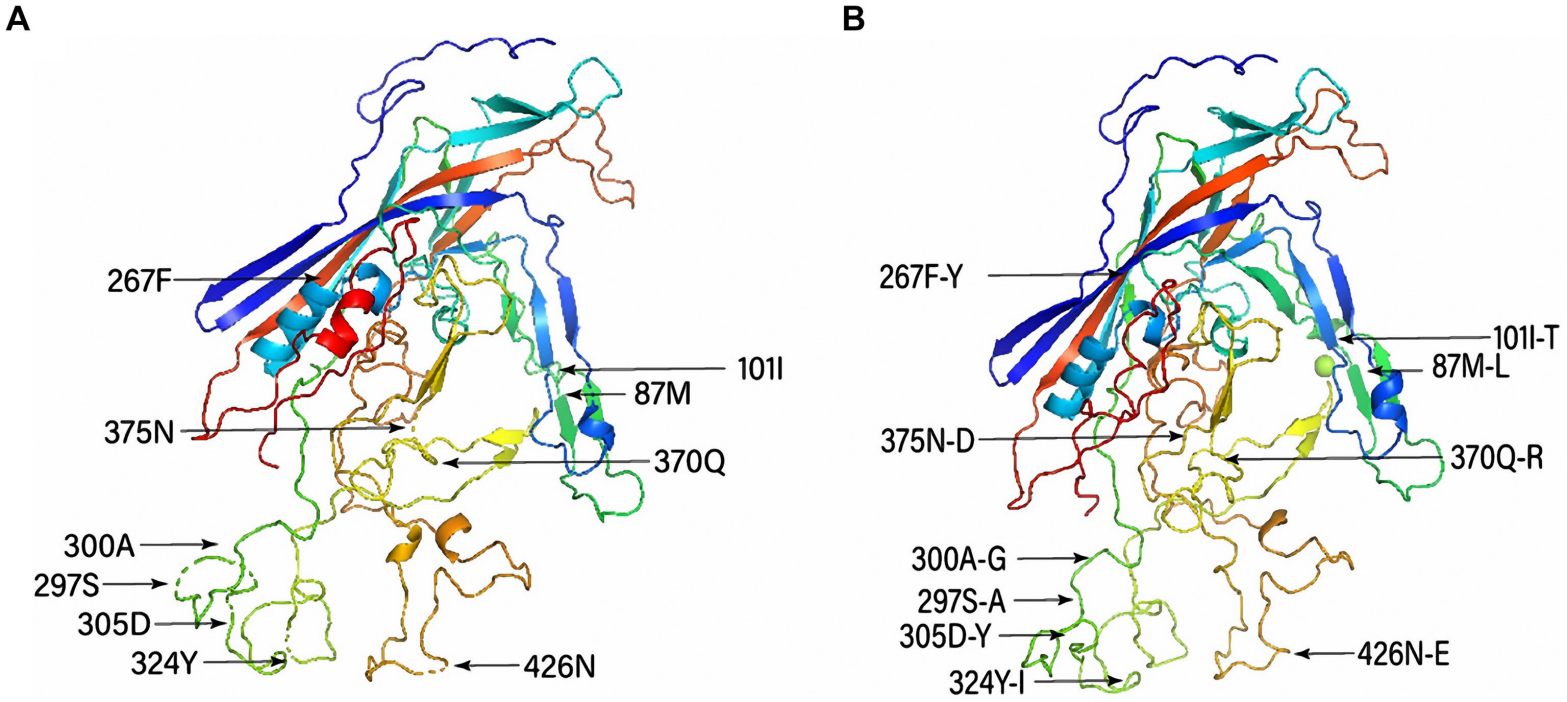
Tertiary structural model of canine parvovirus (CPV) capsid protein (VP2) and distribution of the main mutated amino acid residues. **(A)** Conserved structure of VP2 and **(B)** structure of the mutant VP2 protein.

The representative mutated aa residues of the obtained FPV strains are listed in Supplementary Table S4. Six FPV strains obtained in this study (HN1801, HN1806, HN1906, HN1908, HN1909, and AH2010) had specific Ser91Ala mutations relative to the reference FPV strain (F-E strain, accession number: OL547734), and discontinuous mutations between sites 283 and 310 were only observed in the HB2003 strain. Additionally, the representative mutated aa residues of the obtained CPV-2 strains are presented in Supplementary Table S5. Overall, Met87Leu, Ile101Thr, Phe267Tyr, Ser297Ala, Ala300Gly, Asp305Tyr, Tyr324Ile, and Asn375Asp mutations were detected in the 11 CPV-2 strains obtained in this study. Moreover, the aa mutations ALa5Gly, Gln370Arg, and Asn426Glu were identified in the nine CPV-2c strains (HN1911, JS1901, JS190, HN1907, AH2008, JS2101, HN2103, HN2104, and HN2201). The tertiary structural model of VP2 revealed that Met87Leu, Ile101Thr, and Asn426Glu mutations caused tertiary structural changes in CPV-2 (Figure 4).

## 4. Discussion

In this study, domestic cats with suspected FPV/CPV-2 infections in Central and Eastern China were evaluated; 37 FPV and 11 CPV-2 strains were detected, indicating that CPV-2 is also widely distributed in domestic cats in China. Additionally, based on our previous study, trends in CPV-2 infections revealed that CPV-2c was the most widespread genotype (15). Considering the complex prevalence and code translation of CPV-2 in dogs in China (25), the genetic variation of CPV-2 in domestic cats requires continuous monitoring.

The obtained FPV strains showed 97.72–99.94% identity, whereas the CPV-2 strains displayed 96.7–99.89% identity. The 11 CPV-2 strains obtained in this study were all from Henan and Jiangsu Provinces. The high similarity of the strains in different regions indicates the high infectivity of CPV-2 with the impact of human activity. To further explore the evolutionary trend in FPV and CPV-2 strains, we performed phylogenetic and recombination analyses. Evolutionary analysis revealed a relationship between FPV and CPV strains. The FPV strains were grouped into three branches in the evolutionary tree, suggesting the emergence of different subtypes. In general, CPV-2 harbors more mutations and evolves more rapidly than FPV (26). The new CPV-2a genotype is still the predominant epidemic genotype of CPV-2 in dogs from some provinces of China (27, 28). The two new CPV-2a strains obtained in this study were significantly different from the other new CPV-2a strains reported abroad, and further research is warranted to expand and determine the evolutionary trend. The results of CPV-2 genotyping were not completely consistent with those of phylogenetic analysis, which showed that changes in a single base do not affect the overall evolutionary characteristics of CPV-2.

To understand the evolutionary pathways of new strains, we further analyzed and predicted the recombination possibilities of the obtained and reference strains. Recombination analysis revealed that complex recombination events might occur between FPV and CPV-2 in cats. The HN2103 strain (CPV-2c type) was a recombinant of the FPV strain HN1806 (collected in 2018) and the CPV-2c strain AH2008 (collected in 2020), with AH2008 also being a recombinant of the JS1901 (a CPV-2c type strain collected in 2019) and BM-(11) (a new CPV-2b type strain collected in 2011) strains. The presence of recombinants between the FPV and CPV-2 strains with different genotypes in different regions or years suggests the role of cats as reservoirs for CPV-2 transmission. Moreover, the Chinese CPV-2c strain JS1901 (collected in 2019) and the FPV strain HB2003 (collected in 2020) were the recombinant precursors of the Vietnamese FPV strain F8 (collected in 2018), demonstrating the complex evolution and recombination between the FPV and CPV-2 strains. Furthermore, the Ser297Ala mutation in the antigenic epitope of VP2 protein was observed in all 11 CPV-2 strains isolated from domestic cats in this study. This mutation has been proven to be related to antigenicity changes in new CPV-2a/2b and may have a pronounced effect on continuous host adaptation (29). However, it remains unclear whether the 11 feline CPV-2 strains originated from antibody pressure in vaccinated animals and underwent key point mutations to develop feline tropism. For the prevention and control of CPV-2 in the same residence, the cross-species transmission between cats and dogs may be a challenge (30).

Moreover, the tertiary structure of the VP2 protein of CPV-2 showed that mutations (Met87Leu, Ile101Thr, and Asn426Glu) in the antigenic region may cause structural changes in the VP2 protein, which may result in cross-host transmission (31). Taken together, mutations in the VP2 protein may be responsible for the pathogenicity and evolution of FPV and CPV-2 (32).

In conclusion, our study identified 48 parvovirus strains and confirmed the cocirculation of FPV, new CPV-2a, and CPV-2c strains in domestic cats from Central and Eastern China. This study contributes to the research on the evolution and monitoring of parvovirus in domestic cats, indicating that continuous surveillance may help update the currently available vaccines.

## Data availability statement

The datasets presented in this study can be found in online repositories. The names of the repository/repositories and accession number(s) can be found at: https://www.ncbi.nlm.nih.gov/genbank/, OQ868522-OQ868569.

## Ethics statement

The animal study was reviewed and approved by Animal Welfare and Ethics Committee of Nanyang Normal University (No. 14027). Written informed consent was obtained from the owners for the participation of their animals in this study.

## Author contributions

JJ and XX: conceptualization. SP: methodology and writing—original draft preparation. SP, RJ, and GG: software. XX, JJ, and LY: validation. JJ and YK: formal analysis. SP and GG: investigation. QX and YB: resources. SP and RJ: data curation. JJ and YK: writing—review and editing. SP and RJ: visualization. SP and XX: supervision. LY and YK: project administration. LY: funding acquisition. All authors contributed to the article and approved the submitted version.

## Funding

This study was supported by the National Natural Science Foundation of China (Grant no. 31870917), the Program for Science & Technology Innovation Talents in Universities of Henan Province (Grant no. 22HASTIT042), the program for Innovative Research Team of Science and Technology in University of Henan Province (Grant no. 20IRTSTHN024), and the Guangdong Basic and Applied Basic Research Foundation (Grant no. 2019A1515012006).

## Conflict of interest

The authors declare that the research was conducted in the absence of any commercial or financial relationships that could be construed as a potential conflict of interest.

## Publisher’s note

All claims expressed in this article are solely those of the authors and do not necessarily represent those of their affiliated organizations, or those of the publisher, the editors and the reviewers. Any product that may be evaluated in this article, or claim that may be made by its manufacturer, is not guaranteed or endorsed by the publisher.

## Footnotes

^1^ https://www.chiplot.online

^2^ https://swissmodel.expasy.org-interactive
