# Supplementary material for: Molecular characterization and genetic diversity of parvoviruses prevalent in cats in Central and Eastern China from 2018 to 2022
Source: Front Vet Sci. 2023 Jul 31;10:1218810. doi: 10.3389/fvets.2023.1218810 (PMC10434225; doi:10.3389/fvets.2023.1218810)
Supplement: Supplementary file 1 [file Table_1.docx]

Supplementary Material

**Molecular Characterization and Genetic Diversity of Parvoviruses Prevalent in Cats in Central and Eastern China from 2018 to 2022**

Shunshun Pan^1^, Ruiqi Jiao^1^, Xin Xu^1^, Jun Ji^1,*^, Ge Guo^1^, Lunguang Yao^1^, Yunchao Kan^1^, Qingmei Xie^2^ and Yingzuo Bi^2^

**Correspondence:** Corresponding Author: jijun020@126.com

# Supplementary Table 1. Clinical information on the sources of Chinese parvoviruses isolated in this study

| **Strains** | **Accession no.** | **Genotype** | **Region** | **Date of collection** | **Vaccination record** | **Age** |
| --- | --- | --- | --- | --- | --- | --- |
| HN1801 | OQ868533 | FPV | Henan | Oct. 31, 2018 | 1 dose | 28 d |
| HN1802 | OQ868534 | FPV | Henan | Oct. 31, 2018 | 1 dose | 2 m |
| HN1803 | OQ868535 | FPV | Henan | Oct. 31, 2018 | 2 doses | 3 m |
| HN1804 | OQ868536 | FPV | Henan | Dec. 11, 2018 | N.I.^a^ | 4 m |
| HN1805 | OQ868537 | FPV | Henan | Dec. 11, 2018 | 2 doses | 4 m |
| HN1806 | OQ868538 | FPV | Henan | Dec. 11, 2018 | 1 dose | 2 m |
| HN1901 | OQ868539 | FPV | Henan | Mar. 8, 2019 | 1 dose | 5 m |
| HN1902 | OQ868540 | FPV | Henan | Mar. 12, 2019 | 2 doses | 5 m |
| HN1903 | OQ868541 | FPV | Henan | Mar. 12, 2019 | N.I. | 3 m |
| HN1904 | OQ868542 | FPV | Henan | Apr. 15, 2019 | unvaccinated | 2 m |
| HN1905 | OQ868543 | FPV | Henan | Jun. 14, 2019 | 2 doses | 3 m |
| HN1906 | OQ868544 | FPV | Henan | Jun. 16, 2019 | 1 dose | 2 m |
| HN1907 | OQ868525 | CPV-2c | Henan | Jun. 16, 2019 | 2 doses | 4 m |
| HN1908 | OQ868545 | FPV | Henan | Sep. 1, 2019 | N.I. | 5 m |
| HN1909 | OQ868546 | FPV | Henan | Sep. 1, 2019 | 2 doses | 4 m |
| HN1910 | OQ868547 | FPV | Henan | Oct. 18, 2019 | 1 dose | 3 m |
| HN1911 | OQ868522 | CPV-2c | Henan | Oct. 18, 2019 | 2 doses | 4 m |
| JS1901 | OQ868523 | CPV-2c | Jiangsu | Oct. 18, 2019 | 1 dose | 3 m |
| JS1902 | OQ868524 | CPV-2c | Jiangsu | Oct. 27, 2019 | 1 dose | 1 m |
| AH1901 | OQ868550 | FPV | Anhui | Dec. 1, 2019 | unvaccinated | 4 m |
| AH1902 | OQ868551 | FPV | Anhui | Dec. 1, 2019 | 2 doses | 4 m |
| AH1903 | OQ868552 | FPV | Anhui | Dec. 1, 2019 | 2 doses | 5 m |
| AH1904 | OQ868553 | FPV | Anhui | Dec. 1, 2019 | 2 doses | 3 m |
| AH2001 | OQ868554 | FPV | Anhui | Mar. 1, 2020 | 1 dose | 1 m |
| AH2002 | OQ868555 | FPV | Anhui | Mar. 11, 2020 | 2 doses | 4 m |
| AH2003 | OQ868556 | FPV | Anhui | Mar. 12, 2020 | 2 doses | 5 m |
| AH2004 | OQ868557 | FPV | Anhui | Mar. 12, 2020 | N.I. | 1 m |
| AH2005 | OQ868526 | New CPV-2a | Anhui | Mar. 12, 2020 | 1 dose | 1 m |
| AH2006 | OQ868558 | FPV | Anhui | May. 31, 2020 | 1 dose | 3 m |
| HB2001 | OQ868548 | FPV | Hubei | Jun. 7, 2020 | 2 doses | 4 m |
| HB2002 | OQ868549 | FPV | Hubei | Jun. 7, 2020 | 1 dose | 2 m |
| HB2003 | OQ868569 | FPV | Hubei | Jun. 7, 2020 | unvaccinated | 2 m |
| AH2007 | OQ868559 | FPV | Anhui | Sep. 9, 2020 | 2 doses | 5 m |
| AH2008 | OQ868527 | CPV-2c | Anhui | Sep. 9, 2020 | 2 doses | 6 m |
| AH2009 | OQ868560 | FPV | Anhui | Sep. 9, 2020 | 2 doses | 3 m |
| AH2010 | OQ868563 | FPV | Anhui | Sep. 9, 2020 | unvaccinated | 24 d |
| AH2011 | OQ868561 | FPV | Anhui | Sep. 9, 2020 | 2 doses | 4 m |
| AH2012 | OQ868564 | FPV | Anhui | Sep. 9, 2020 | 2 doses | 5 m |
| JS2001 | OQ868562 | FPV | Jiangsu | Aug.31,2020 | 1 dose | 5 m |
| JS2002 | OQ868565 | FPV | Jiangsu | Aug.31,2020 | N.I. | 4 m |
| JS2101 | OQ868528 | CPV-2c | Jiangsu | May.17, 2021 | 2 doses | 5 m |
| HN2101 | OQ868566 | FPV | Henan | Jun. 20, 2021 | 1 dose | 4 m |
| HN2102 | OQ868529 | New CPV-2a | Henan | Jun. 20, 2021 | 1 dose | 3 m |
| HN2103 | OQ868531 | CPV-2c | Henan | Jun. 20, 2021 | 2 doses | 5 m |
| HN2104 | OQ868530 | CPV-2c | Henan | Jun. 20, 2021 | 2 doses | 5 m |
| HN2105 | OQ868567 | FPV | Henan | Jun. 20, 2021 | 1 dose | 3 m |
| JS2201 | OQ868568 | FPV | Jiangsu | Apr. 4, 2022 | 1 dose | 2 m |
| HN2201 | OQ868532 | CPV-2c | Henan | Jun. 3, 2022 | unvaccinated | 5 m |

^a^ no information

# Supplementary Table 2. Reference strains used in the study

| **Strains** | **Accession no.** | **Genotype** | **Region** | **Year of collection** |
| --- | --- | --- | --- | --- |
| F-E | OL547734 | FPV | China | 2020 |
| F-D34 | OL547735 | FPV | China | 2019 |
| 933-07 | EU360958 | FPV | Hungary | 2007 |
| 10786 | HQ184200 | FPV | South Korea | 2008 |
| PT015-08 | KU248463 | FPV | Portugal | 2008 |
| PT006-06 | KU248461 | FPV | Portugal | 2006 |
| Felocell11 | EU498681 | FPV | Italy | 2008 |
| PT264-14 | KT240135 | FPV | Portugal | 2014 |
| PT083-13 | KT240133 | FPV | Portugal | 2013 |
| PT022-08 | KT240131 | FPV | Portugal | 2008 |
| PT001-07 | KT240129 | FPV | Portugal | 2007 |
| PT020-06 | KT240128 | FPV | Portugal | 2006 |
| Rac2.2-78 | JN867595 | FPV | The USA | 1978 |
| BJ-22 | FJ231389 | FPV | China | 2008 |
| FPV-b CU4 | M24004 | FPV | The USA | 1970 |
| Giant panda | MZ322607 | FPV | China | 2018 |
| F1 | MT857283 | FPV | Viet Nam | 2018 |
| F17 | MT857280 | FPV | Viet Nam | 2019 |
| F11 | MT857274 | FPV | Viet Nam | 2019 |
| F9 | MT857272 | FPV | Viet Nam | 2018 |
| F8 | MT857271 | FPV | Viet Nam | 2018 |
| F7 | MT857270 | FPV | Viet Nam | 2018 |
| F-D33 | OL547736 | FPV | China | 2019 |
| CO-952-10 | JX475245 | FPV | The USA | 2010 |
| CU-4 | M38246 | FPV | The USA | 1990 |
| 790312 | M38245 | CPV-2 | The USA | 1990 |
| CPVint (vaccine) | FJ197846 | CPV-2 | South Korea | 2007 |
| Pfizer-vaccine-06 | EU914139 | CPV-2 | The USA | 2008 |
| CPV-15 | M24003 | CPV-2a | The USA | 1989 |
| isolate 39 | M74849 | CPV-2b | Italy | 1995 |
| CPV-339 | AY742933 | new CPV-2a | The USA | 2004 |
| K022 | EU009203 | new CPV-2a | South Korea | 2007 |
| CPV-LZ2 | JQ268284 | new CPV-2b | China | 2011 |
| CPV-BM(11) | JQ743894 | new CPV-2b | China | 2012 |
| LCPV V204 | AB054221 | new CPV-2b | Japan | 2000 |
| G367-97 | FJ005202 | CPV-2c | Germany | 1997 |
| 2c-ME28-ECU2012 | KF149984 | CPV-2c | Ecuador | 2012 |
| G333-99 | FJ005204 | CPV-2c | Germany | 1999 |
| F2016009 | MH329283 | new CPV-2a | China | 2016 |
| F2016010 | MH329284 | new CPV-2a | China | 2016 |
| F2016015 | MH329285 | new CPV-2a | China | 2016 |

# Supplementary Table 3. Antigen site prediction of VP2 proteins

| **Amino acid sites** | **Amino acid sequence** | **Score** |
| --- | --- | --- |
| 247–256 | ENSVPVHLLR | 1.207 |
| 145-156 | IFNVVLKTVSES | 1.179 |
| 479-493 | KPRLHVNAPFVCQNN | 1.168 |
| 495-505 | PGQLFVKVAPN | 1.166 |
| 100-113 | DTHVQIVTPWSLVDA | 1.135 |
| 135-143 | ELHLVSFEQ | 1.134 |
| 267-276 | YFDCKPCRLT | 1.128 |
| 531-542 | GKLVFKAKLRAS | 1.128 |
| 453-466 | PLTALNNVPPVYPN | 1.128 |
| 80-85 | KPVVVN | 1.126 |
| 66-72 | SRLVHLN | 1.121 |
| 267-276 | LGLPPFLNSL | 1.120 |
| 170-178 | TASLMVALD | 1.119 |
| 285-294 | LGLPPFLNSL | 1.112 |
| 421-434 | EVGYSAPYYSFE | 1.103 |
| 335-346 | EVGYSAPYYSFE | 1.086 |
| 569-581 | MKIVYEKSQLAPR | 1.078 |
| 398-403 | FTYIAH | 1.072 |
| 126-131 | WQLIVN | 1.062 |
| 217-222 | TLIPSH | 1.038 |
| 230-235 | TNVYHG | 1.038 |
| 558-565 | QFNYVPNN | 1.037 |
| 195-202 | LGFYPWKP | 1.207 |

# Supplementary Table 4. VP2 amino acid mutation sites compared with the FPV reference strain (F-E, GenBank accession number: OL547734)

| Strains | Amino acid mutation sites | | | | | | |
| --- | --- | --- | --- | --- | --- | --- | --- |
|  | 5 | 91 | 113 | 191 | 299 | 338 | 346 |
| OL547734 | A | S | D | R | G | Y | E |
| OL57735 | - | - | - | - | - | - | - |
| EU360958 | - | - | - | - | - | - | - |
| HQ184200 | - | A | - | - | - | - | - |
| KU248463 | - | A | - | - | - | - | - |
| KU248461 | - | A | - | - | - | - | - |
| EU498681 | - | A | - | - | - | - | - |
| KT240135 | - | A | - | - | - | - | - |
| KT240133 | - | A | - | - | - | - | - |
| KT240131 | - | A | - | - | - | - | - |
| KT240129 | - | A | - | - | - | - | - |
| KT240128 | - | A | - | - | - | - | - |
| JN867595 | - | A | - | - | - | - | - |
| FJ231389 | - | A | - | - | - | - | - |
| M24004 | - | A | - | - | - | - | - |
| MZ322607 | - | A | - | - | - | - | - |
| MT857283 | - | A | - | - | - | - | - |
| MT857280 | - | A | - | - | - | - | - |
| MT857274 | - | A | - | - | - | - | - |
| MT857272 | - | A | - | - | - | - | - |
| MT857271 | - | A | - | - | - | - | - |
| MT857270 | - | A | - | - | - | - | - |
| OL547736 | - | - | - | - | - | - | N |
| JX475245 | - | A | - | - | - | - | - |
| M38246 | - | A | - | - | - | - | - |
| HN1801 | G | A | - | - | - | - | - |
| HN1802 | - | - | - | - | - | - | - |
| HN1803 | - | - | - | - | - | - | - |
| HN1804 | - | - | - | - | E | - | - |
| HN1805 | - | - | - | - | - | H | - |
| HN1806 | - | A | - | - | - | - | - |
| HN1901 | - | - | G | - | - | - | - |
| HN1902 | - | - | - | - | - | - | - |
| HN1903 | - | - | - | - | - | - | - |
| HN1904 | - | - | - | K | - | - | - |
| HN1905 | - | - | - | - | R | - | - |
| HN1906 | - | A | - | - | - | - | - |
| HN1908 | - | A | - | - | - | - | - |
| HN1909 | - | A | - | - | - | - | - |
| HN1910 | - | - | - | - | - | - | - |
| HB2001 | - | - | - | - | - | - | - |
| HB2002 | - | - | - | - | - | - | - |
| AH1901 | - | - | - | G | - | - | - |
| AH1902 | - | - | - | G | - | - | - |
| AH1903 | - | - | - | - | R | - | - |
| AH1904 | - | - | - | - | - | - | - |
| AH2001 | - | - | - | - | - | - | - |
| AH2002 | - | - | - | - | - | - | - |
| AH2003 | - | - | - | - | - | - | - |
| AH2004 | - | - | - | - | - | - | - |
| AH2006 | - | A | - | - | - | - | - |
| HB2003 | - | - | - | - | - | - | - |
| AH2007 | - | - | - | - | - | - | - |
| AH2009 | - | - | - | - | - | - | - |
| AH2011 | - | - | - | - | - | - | - |
| JS2001 | - | - | - | - | - | - | - |
| AH2010 | - | - | - | - | - | - | - |
| AH2012 | - | - | - | - | - | - | - |
| JS2002 | - | - | - | - | - | - | - |
| HN2101 | - | - | - | - | - | - | - |
| HN2105 | - | - | - | - | - | - | G |
| JS2201 | - | - | - | - | - | - | - |

“-” represents the site of obtained strain was the same as reference strain. The underlined strains are Chinese FPV strains obtained in this study (A: Ala, D: Asp, E: Glu, I: Ile, G: Gly, K: Lys, M: Met, N: Asn, R: Arg, S: Ser, T: Thr, Y: Tyr).

# Supplementary Table 5. VP2 aa mutation sites compared to the CPV-2 reference strain (790312, GenBank accession number: M38245)

| Strains | Amino acid mutation sites | | | | | | | | | | | | | | |
| --- | --- | --- | --- | --- | --- | --- | --- | --- | --- | --- | --- | --- | --- | --- | --- |
|  | 5 | 13 | 87 | 101 | 267 | 297 | 300 | 305 | 324 | 370 | 375 | 426 | 440 | 447 | 527 |
| M38245 | A | P | M | I | F | S | A | D | Y | Q | N | N | T | I | F |
| FJ197846 | - | - | - | - | - | - | - | - | - | - | N | - | - | - | - |
| EU914139 | - | - | - | - | - | - | - | - | - | - | D | - | - | - | - |
| M24003 | - | - | L | T | - | - | G | Y | - | - | D | - | - | - | - |
| M74849 | - | - | L | T | - | - | G | Y | - | - | D | D | - | - | - |
| AY742933 | - | - | L | T | - | A | G | Y | - | - | D | - | - | - | - |
| EU009203 | - | - | L | T | - | A | G | Y | - | - | D | - | A | - | - |
| JQ268284 | - | - | L | T | Y | A | G | Y | - | - | D | D | A | - | - |
| JQ743894 | - | - | L | T | Y | A | G | Y | - | - | D | D | A | - | - |
| AB054221 | - | - | L | T | - | A | G | Y | - | - | D | D | A | - | - |
| FJ005202 | - | - | L | T | - | A | G | Y | - | - | D | E | - | - | - |
| KF149984 | - | - | L | T | - | A | G | Y | - | - | D | E | - | - | - |
| FJ005204 | - | - | L | T | - | A | G | Y | - | - | D | E | - | - | - |
| MH329283 | - | - | L | T | Y | A | G | Y | - | - | D | - | A | - | - |
| MH329284 | - | - | L | T | Y | A | G | Y | - | - | D | - | A | - | - |
| MH329285 | - | - | L | T | Y | A | G | Y | - | - | D | - | A | - | - |
| HN1911 | G | - | L | T | Y | A | G | Y | I | R | D | E | - | - | - |
| JS1901 | G | - | L | T | Y | A | G | Y | I | R | D | E | - | - | L |
| JS1902 | G | - | L | T | Y | A | G | Y | I | R | D | E | - | - | - |
| HN1907 | G | S | L | T | Y | A | G | Y | I | R | D | E | - | - | - |
| AH2005 | - | - | L | T | Y | A | G | Y | I | - | D | - | A | - | - |
| AH2008 | G | - | L | T | Y | A | G | Y | I | R | D | E | - | M | - |
| JS2101 | G | - | L | T | Y | A | G | Y | I | R | D | E | - | - | - |
| HN2102 | - | - | L | T | Y | A | G | Y | I | - | D | - | A | - | - |
| HN2104 | G | - | L | T | Y | A | G | Y | I | R | D | E | - | - | - |
| HN2103 | G | S | L | T | Y | A | G | Y | I | R | D | E | - | - | - |
| HN2201 | G | - | L | T | Y | A | G | Y | I | R | D | E | - | - | - |

“-” represents the site of obtained strain was the same as reference strain. The underlined strains are Chinese CPV-2 strains obtained in this study (A: Ala, D: Asp, E: Glu, F: Phe, I: Ile, G: Gly, L: Leu, M: Met, N: Asn, P: Pro, Q: Gln, R: Arg, S: Ser, T: Thr, Y: Tyr).
